# Editorial: New insights in neurodegeneration: highlights from the 42nd Annual Meeting of the Australasian Neuroscience Society (Perth, Western Australia)

**DOI:** 10.3389/fnins.2026.1954339

**Published:** 2026-08-27

**Authors:** Sarah C. Hellewell, Stuart I. Hodgetts, Samantha L. Gardner, Hamid R. Sohrabi

**Affiliations:** 1 Faculty of Health Sciences, Curtin Medical Research Institute, Curtin University, Bentley, WA, Australia; 2 The Perron Institute for Neurological and Translational Science, Nedlands, WA, Australia; 3 Centre for Neuromuscular and Neurological Disorders, University of Western Australia, Crawley, WA, Australia; 4 School of Human Sciences, University of Western Australia, Crawley, WA, Australia; 5 School of Medical and Health Sciences, Edith Cowan University, Joondalup, WA, Australia; 6 Alzheimer's Research Australia, Ralph and Patricia Sarich Neuroscience Research Institute, Nedlands, WA, Australia; 7 The University of Western Australia Medical School, Crawley, WA, Australia; 8 Centre for Healthy Ageing, Health Futures Institute, Murdoch University, Murdoch, WA, Australia; 9 School of Psychology, Murdoch University, Murdoch, WA, Australia

**Keywords:** Alzheimer's disease, cognitive impairment, gray matter (also, grey matter), neurodegeneration, neurosyphilis, oxysterol, traumatic brain injury

Neurodegenerative disease has an expanding burden in a modern society (1), with an aging global population and increasing disease prevalence (2). The 42nd Annual Meeting of the Australasian Neuroscience Society (ANS), held in Perth, Western Australia in December 2024, brought together researchers working across diverse aspects of neuroscience, with a particular focus on neurodegeneration. This Research Topic presents five articles that collectively articulate a coherent set of themes emerging through this meeting and more broadly in the field: the hippocampus as a site of early neural compromise; neuroinflammation as a shared pathological driver across mechanistically distinct conditions; synaptic dysfunction as a link between molecular dysfunction and cognitive dysfunction; and application of multimodal *in vivo* brain imaging as a means to detect (sub)clinical pathology, monitor progression and give theragnostic insight.

The hippocampus occupies a central position across all papers in this Research Topic. Han et al. used proton magnetic resonance spectroscopy (1H-MRS) to quantify hippocampal cellular metabolism in asymptomatic neurosyphilis (ANS). Despite the absence of overt neurological symptoms and comparable Montreal Cognitive Assessment scores between symptomatic and asymptomatic patients and cognitively unimpaired, ANS patients had significantly reduced N-acetylaspartate-to-creatine (NAACr) ratios in the bilateral hippocampi, indicating a subclinical metabolic signature of reduced neuronal integrity that preceded detectable clinical or structural change. These ratios correlated positively with cognitive scores and inversely with CSF white blood cell count and protein levels, suggesting a link between neuroinflammation and hippocampal neurometabolic alterations. High-dose penicillin therapy normalized bilateral NAA/Cr ratios to levels indistinguishable from cognitively unimpaired, demonstrating that this metabolic disturbance is reversible with current treatment measures. More broadly, this work demonstrates the power of neurometabolic imaging to reveal subclinical neuropathology in infectious CNS diseases, where conventional assessments may fail to capture the true extent of neuronal alteration.

Using a controlled cortical impact mouse model of moderate traumatic brain injury (TBI), Pan et al. investigated whether the calcium channel protein transient receptor potential canonical 1 (TRPC1) plays a role in the development of post-TBI depression and its pathogenesis via modulation of the immune response. Four weeks following controlled cortical impact TBI, mice showed significant depressive- and anxiety-like behaviors. TRPC1 expression was downregulated in the hippocampus at this time, accompanied by reduced synaptic proteins, elevated pro-inflammatory cytokines, and increased reactive astrocytes and microglia. Importantly, Adeno-associated virus (AAV)-mediated TRPC1 overexpression attenuated neuroinflammation, restored synaptic function, and ameliorated depressive-like behaviors in TBI mice, identifying TRPC1 as a novel therapeutic target for post-TBI neuropsychiatric sequelae. These findings position TRPC1 as a mechanistic bridge between post-traumatic neuroinflammation and psychiatric outcomes, opening a new avenue for targeted intervention in the significant proportion of TBI survivors who develop depression.

The comprehensive review of Ricci et al. examines the role of oxysterols (oxidized derivatives of cholesterol) in CNS physiology of disease, illuminating the underappreciated role of these oxysterols as both physiological regulators and pathological drivers of neurodegeneration. Authors detail how enzymatically produced oxysterols generated in neurons and glia support cholesterol homeostasis, gene expression, and synaptic plasticity under normal conditions, while oxidative-stress-derived species accumulate in disease states to drive neuroinflammation, mitochondrial dysfunction, and cell death. A particular strength of this review is its integration of oxysterol biology across multiple cell types and neurotransmitter systems: authors trace how disrupted oxysterol signaling impairs glutamatergic, GABAergic, cholinergic, dopaminergic, and serotonergic function, linking systemic lipid metabolism to the chronic neuroinflammatory cascades that characterize Alzheimer's disease (AD), Parkinson's disease, multiple sclerosis, and neuropsychiatric disorders. By positioning oxysterols not merely as downstream markers of neurodegeneration but as active upstream contributors to glial dysfunction, synaptic failure, and myelin pathology, this work identifies oxysterol metabolism as a promising therapeutic target and highlights the potential of specific oxysterols as biomarkers of neuroaxonal integrity.

Wu et al. investigated the neurobiological mechanisms underlying the cognitive benefits of moxa-combustion byproducts in a mouse model of AD. Building on the well-established but underexplored link between olfactory dysfunction and early AD pathology (3), the authors demonstrate that 12 weeks of inhaled moxa-combustion byproducts in APP/PS1 transgenic mice significantly improved spatial learning, memory, and olfactory performance, while also reducing anxiety- and depression-like behaviors. Treatment increased mitral cell numbers in the olfactory bulb and neuronal density in the hippocampal CA1, enhanced synaptic ultrastructure, and modulated the GSK-3β/CREB signaling pathway. Critically, these effects were abolished by olfactory blockade using 3-methylindole, providing evidence that the therapeutic action of moxa-combustion byproducts is olfactory-mediated rather than systemic. This work offers a novel mechanistic framework for understanding how olfactory stimulation may engage neuroprotective signaling pathways relevant to AD progression, and raises the broader question of whether the olfactory system represents an underutilized therapeutic entry point for neurodegenerative disease.

In their manuscript, Kurth et al. address the longstanding question of how hemispheric asymmetry in gray matter changes across the adult lifespan. In a longitudinal sample of over 2,300 middle-aged to older adults scanned twice approximately 2.4 years apart as part of the UK Biobank, authors mapped voxel-wise changes in gray matter asymmetry over time, finding that asymmetry most notably shifted in the temporal and occipital lobes and the cerebellum. Decreases in asymmetry were more common than increases overall, though with an intriguing directional pattern: leftward asymmetries tended to diminish while rightward asymmetries tended to grow. Importantly, these changes were not significantly associated with chronological age or biological sex, suggesting that the trajectory of asymmetry change across this age range is relatively uniform rather than accelerating with age or differing between males and females. This study provides valuable large-scale longitudinal evidence that gray matter asymmetry is a dynamic rather than fixed neuroanatomical property, with implications for understanding both normal aging and the structural brain changes that characterize neurodegenerative disease.

Together, this Research Topic of papers reflect the breadth and vitality of neurodegeneration research presented at the 42nd ANS Annual Meeting. These works exemplify the idea that neurodegeneration is rarely a discrete event, but rather the cumulative product of converging molecular insults that unfold across time and are detectable, often subclinically, long before clinical thresholds are reached. The modifiable nature of pathology highlighted in several of these papers suggests that with earlier detection and targeted intervention there may be a future in which we can alter the trajectory of neurodegenerative disease.

